# Impact of Class III Obesity (Morbid Obesity) on the Perioperative, Functional, and Oncological Outcomes of Robotic-Assisted Radical Prostatectomy

**DOI:** 10.3390/cancers17040709

**Published:** 2025-02-19

**Authors:** Abdel Rahman Jaber, Shady Saikali, Marcio Covas Moschovas, Ahmed Gamal, Ela Patel, Marco Sandri, Travis Rogers, Vipul Patel

**Affiliations:** 1 Adventhealth Global Robotics Institute, Celebration, FL 34747, USA; 2 Urology Department, University of Central Florida (UCF), Orlando, FL 32816, USA; 3 Big and Open Data Innovation Laboratory (BODaI-Lab) and Data Methods and Systems Statistical, 25122 Brescia, Italy

**Keywords:** class III obesity, morbid obesity, radical prostatectomy, robotic-assisted

## Abstract

**Background:** The growing prevalence of obesity, particularly morbid obesity (BMI > 40 kg/m^2^), presents unique challenges in surgical management. In robotic-assisted radical prostatectomy (RARP) for prostate cancer, morbid obesity is associated with longer operative times, increased blood loss, and complex nerve-sparing procedures. These challenges may impact functional and oncological outcomes, making it essential to understand how high BMI affects RARP results. However, data on outcomes for patients with morbid obesity remain limited. **Methods:** We retrospectively reviewed 14,444 patients who underwent RARP by a single surgeon from 2008 to 2023. A total of 183 patients with morbid obesity (BMI > 40 kg/m^2^) were matched 1:1 with 183 normal-weight patients (BMI 18.5–24.9 kg/m^2^) using propensity-score matching based on age, PSA levels, clinical staging, ISUP grade, comorbidities, and baseline urinary and sexual function scores. We compared perioperative variables, complications, and oncological and functional outcomes between groups. **Results:** Patients with morbid obesity had longer console times (median 90 vs. 75 min, *p* < 0.001) and higher rates of estimated blood loss over 100 mL (39% vs. 22%, *p* = 0.001). Full nerve-sparing was less frequent in this group (16.9% vs. 38.8%, *p* < 0.001), and postoperative lymphocele formation rates were higher (12.7% vs. 6.0%, *p* = 0.021). While patients with morbid obesity demonstrated a higher incidence of pT3a disease (34.4% vs. 20.2%, *p* = 0.015), no significant differences were observed in BCR and PSM (24% vs. 18%, *p* = 0.25) or functional outcomes, such as potency and urinary continence, at 12 and 24 months after operation. **Conclusions:** RARP in patients with morbid obesity is safe and feasible, with low rates of complications and no observed differences in functional and oncological outcomes compared to normal-weight patients. Further studies are needed to assess long-term outcomes in this growing patient population.

## Simple Summary

This study highlights the growing impact of the global rise in obesity and morbid obesity on patients undergoing robotic-assisted radical prostatectomy (RARP), addressing the unique challenges posed by this high-risk population. By analyzing the largest cohort of patients with morbid obesity reported in the literature, it provides comprehensive data on intraoperative, functional, and oncological outcomes. These findings offer valuable insights to guide future research and support informed clinical decision-making, aiding in the optimization of surgical interventions for patients with obesity and morbid obesity.

## 1. Introduction

Prostate cancer is the second most common malignancy affecting men worldwide [1]. The introduction of robotic surgical systems, such as the Da Vinci platform, has revolutionized the treatment of localized prostate cancer. Robotic-assisted radical prostatectomy (RARP) has gained widespread popularity due to its advantages, including improved precision, reduced blood loss, shorter recovery times, and lower complication rates compared to open or laparoscopic surgery. As a result, more patients, including those with obesity, are opting for RARP over other treatment modalities, recognizing the potential benefits it offers in complex surgical scenarios.

Obesity, identified by the World Health Organization (WHO) as a global health concern, has seen a significant rise between 1990 and 2022, with the percentage of adults aged 18 and older living with obesity more than doubling from 7% to 16% [2]. Several factors have associated obesity with cancer development. The low exposure to insulin growth factor, lipid levels, and insulin resistance have all been proposed as pathways to explain the association between cancer and obesity [3]. The overlap of obesity and prostate cancer frequently complicates patient management, as both conditions pose unique challenges. The diagnosis of prostate cancer relies on physical examination, PSA testing, imaging, and biopsy, all of which can be influenced by obesity. Obesity can impact the detection of prostate cancer, making early diagnosis more challenging. Increased adiposity may hinder physical examinations, interfere with imaging accuracy, and contribute to lower PSA levels due to reduced androgen synthesis in men with higher BMIs [4]. As a result, patients with obesity may be less likely to be diagnosed at an early stage. This is particularly significant given that most prostate cancer cases are detected through biopsy following an elevated PSA finding.

Obesity presents significant challenges during robot-assisted radical prostatectomy (RARP), particularly due to the steep Trendelenburg position required for the procedure. This position increases the risks of anesthesia-related complications and surgical difficulties [5,6]. Additionally, obesity affects oncological outcomes like biochemical recurrence and prostate cancer-specific mortality [7,8]. However, advancements in robotic surgery have expanded the eligibility for surgical intervention in patients with obesity, enabling more personalized and effective management of prostate cancer in this group.

The impact of obesity on the outcomes of robot-assisted radical prostatectomy (RARP) has been extensively studied, with mixed and conflicting results reported in the literature [9,10,11,12,13,14,15,16,17]. However, there is a notable lack of research examining the effects of class III obesity (BMI > 40 kg/m^2^) on RARP outcomes [18,19,20,21]. In our current study, we aimed to explore the influence of class III obesity on perioperative, functional, and oncological outcomes of RARP.

## 2. Materials and Methods

We reviewed data from 14,444 patients who underwent RARP between 2008 and 2023 at our institution, all performed by a single surgeon (V.P.). The data were collected prospectively and analyzed retrospectively under an IRB-approved protocol with informed consent from all participants. To control for confounding factors, 183 patients with morbid obesity (BMI > 40 kg/m^2^) were matched (1:1 ratio) with 183 normal-weight patients (BMI 18.5–24.9 kg/m^2^) from a pool of 2509 patients. Patients with a history of radiation therapy or suspected preoperative metastasis were excluded (Supplementary Figure S1).

We recorded and analyzed perioperative and postoperative variables, including estimated blood loss (EBL), operative time, postoperative complications, positive surgical margins (PSM) rates, and biochemical recurrence (BCR). Urinary continence was defined as using 0 pads per day (PPD). Additionally, we assessed urinary symptoms using the American Urological Association Symptoms Score (AUASS). Sexual function was evaluated using a scoring system from 1 to 5, where 5 indicated the highest level of satisfaction with achieving and maintaining erections sufficient for penetration, assessed at 3, 12, 18, 24, and 48 months after RARP. The surgical technique used followed protocols established in previous studies from our center [22,23,24,25]. Lymph node dissection was performed for patients with Gleason 7 and above.

### 2.1. Endpoints

Our primary endpoint was to compare and evaluate the impact of obesity class III (morbid obesity) on perioperative performance and complication rates according to the Clavien–Dindo classification. The secondary endpoints assessed the functional and oncological outcomes of RARP.

### 2.2. Propensity Score Matching

To minimize the bias introduced by potential confounders, 183 patients with class III obesity (morbid obesity) were matched with 183 patients from a normal range BMI cohort in a 1:1 ratio (Table 1). The propensity score (PS) was calculated using a multivariable logistic regression model, accounting for variables such as age, PSA levels, clinical T staging, biopsy ISUP grading, comorbidities, history of prostate surgery, the Sexual Health Inventory for Men (SHIM) score, and the American Urological Association symptom score (AUASS).

**Figure 1:**
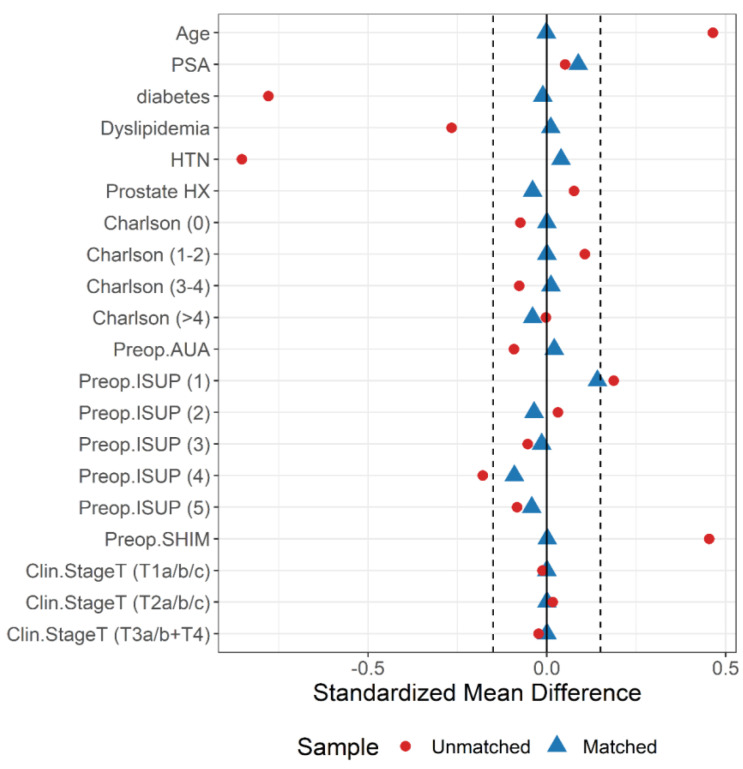
Love plot depicting the distributional balance of preoperative variables before and after propensity score (PS) matching.

Matching was conducted using the nearest-neighbor algorithm with a caliper width of 0.25 times the standard deviation of the logit score, without replacement. The quality of matching was assessed through the standardized mean difference, which indicates the extent of covariate imbalance. Covariates with a standardized difference of less than 0.15 in absolute value were considered adequately balanced. Additionally, we employed the nonparametric Kolmogorov–Smirnov test to evaluate the equality of distributions for continuous variables and Fisher’s exact test for categorical variables. Figure 1 displays a Love plot depicting the balance of variables pre- and post-matching.

### 2.3. Statistical Analysis

Continuous variables were summarized with the median and interquartile range (IQR), while categorical variables were reported as absolute and relative frequencies. Between-group comparisons for continuous and categorical variables were conducted using the Wilcoxon rank-sum and Fisher’s exact tests, respectively. For the analysis of time-to-event outcomes (potency, continence, and BCR) in matched cohorts, Cox regression was applied to estimate hazard ratios with confidence intervals (CIs). Cumulative incidence curves were estimated using the Kaplan–Meier method. A *p*-value of less than 0.05 was considered statistically significant for all analyses. Statistical analyses were conducted using Stata 16.1 (StataCorp, College Station, TX, USA) and R 4.4.1 (R Foundation for Statistical Computing, Vienna, Austria).

## 3. Results

### 3.1. Patient Demographics

Table 1 shows that the two study groups were satisfactorily balanced after matching and exhibited similar preoperative characteristics, including age, PSA, co-morbidities, clinical stage, ISUP, SHIM, and AUA scores. The median follow-up time for both groups was 5 years.

### 3.2. Intraoperative Parameters

There was a statistically significant difference between the two groups regarding EBL and console time. While the median EBL was 100 mL for both groups, 39% of the patients with morbid obesity had an EBL greater than 100 mL compared to 22% in the normal-weight group. The median console time was also significantly longer for the morbidly obese group (90 min vs. 75 min). No immediate intraoperative complications were reported in either group. However, there was a notable difference in the degree of nerve-sparing achieved, with full nerve-sparing performed in 16.9% of the patients with morbid obesity compared to 38.8% of the control group (Table 2).

### 3.3. Postoperative Parameters and Complications

**Figure 2:**
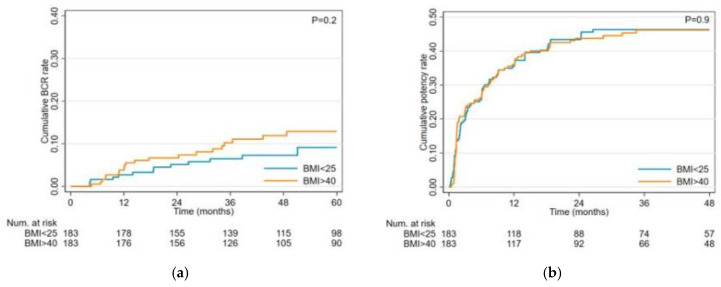

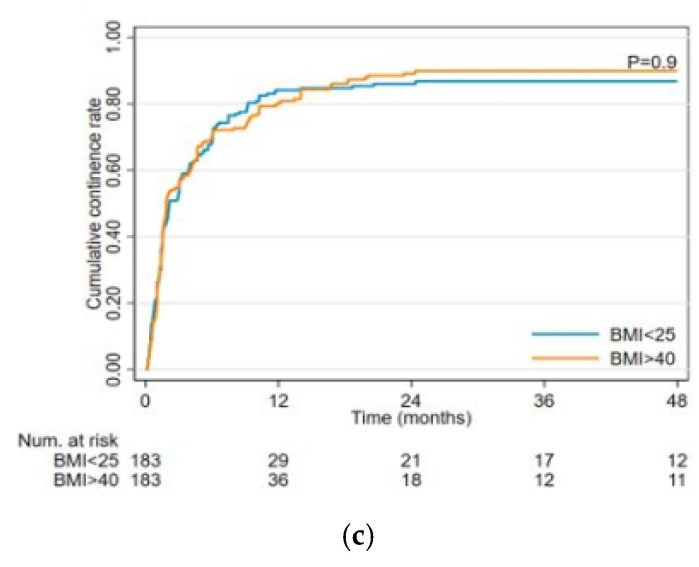
(**a**) Cumulative incidence of biochemical recurrence stratified by BMI; (**b**) Cumulative incidence of potency recovery stratified by BMI; (**c**) Cumulative incidence of Continence stratified by BMI.

We analyzed postoperative complications using the Clavien–Dindo classification and did not find statistically or clinically significant differences between the two groups. However, lymphocele formation was significantly higher in the morbid obesity group compared to the control group (25% vs. 11%) (Table 2). Pathological T staging showed no difference between the two groups overall. Still, within the pT3a category, there was a significant difference, with a higher incidence in the morbid obesity group compared to the control group (34% vs. 20%). Additionally, we did not find significant differences in BCR (Figure 2a) or positive surgical margins (PSM) between the groups (24% vs. 18%, *p* = 0.25) (Table 2). There were no significant differences in cumulative potency rates (Figure 2b) or continence rates (Figure 2c) between the study and control groups. Table 3 shows the cumulative potency, continence, and BCR rates at 12 and 24 months.

## 4. Discussion

Robotic-assisted prostatectomy in patients with morbid obesity poses unique challenges that can affect surgical and oncological outcomes. Previous studies have highlighted that obesity is associated with more aggressive prostate cancer and increased perioperative risks, such as longer operative times and higher blood loss [5,19,20,21]. These factors emphasize the need to understand their impact on intraoperative and postoperative outcomes. Additionally, obesity is associated with unfavorable oncological characteristics, which complicates cancer control and recovery of key functions like potency and continence [8,10,17,26,27,28]. However, there is limited literature specifically addressing patients with morbid obesity (BMI > 40 kg/m^2^). To the best of our knowledge, this study represents the largest cohort in the literature involving patients with a BMI greater than 40, addressing a gap in the research on this group.

Our study demonstrated differences in intraoperative variables such as operative time and estimated blood loss (EBL) in patients with morbid obesity undergoing robotic-assisted prostatectomy. These results stem from excess adipose tissue prolonging Retzius space dissection and limited intraabdominal space complicating the urethrovesical anastomosis. Agarwal et al., who used the extraperitoneal approach, matched 40 patients with morbid obesity with 40 patients without morbid obesity. They observed a mean total operative time of 238 min for patients with morbid obesity compared to 176 min for patients without morbid obesity. The mean EBL was 235 mL in the morbidly obese group compared to 192 mL in the non-morbidly obese group [21]. Haidar et al. found no significant differences in operative times between groups, although the mean EBL was significantly higher in patients with morbid obesity at 129.8 mL compared to 112.5 mL [19]. Yates et al. reported a mean operative time of 163 min in patients with morbid obesity, with an average EBL of 210 mL [20]. Han et al., using a national database, found that patients with morbid obesity experienced higher intraoperative complication rates and blood transfusion rates compared to patients without obesity, with transfusion rates of 2.41% versus 1.52% [18]. These findings indicate longer operative times and higher blood loss among patients with morbid obesity, accompanied by an increased need for intraoperative management.

Obesity is widely recognized in the literature as an independent predictor of lymphocele formation, increasing the rates of both lymphocele occurrence and symptomatic lymphocele [29]. Gobler noted that patients with obesity may face nearly a threefold higher risk of developing symptomatic lymphoceles compared to patients without obesity [30]. This observation aligns with findings from other studies [31,32]. Our results similarly reflect this trend. The higher risk of lymphocele in patients with obesity may stem from increased pelvic adipose tissue, which often has chronic inflammation and pelvic fluid accumulation. This inflammation, exacerbated by surgery and difficult intraoperative conditions, likely contributes to the formation of postoperative lymphocele [30,31]. Despite recent techniques described to minimize lymphoceles, this condition is still challenging in patients with obesity [33].

Oncological outcomes are crucial for RARP. Agarwal et al. found no significant differences in positive surgical margin (PSM) rates or Gleason scores between morbidly obese and non-morbidly obese groups. However, a significantly higher proportion of patients with morbid obesity had pT3 disease (27.5% vs. 7.5%) [21]. Haidar et al. reported a higher incidence of PSMs in the morbidly obese group (25% vs. 11.4%), though this difference only approached statistical significance (*p* = 0.097) [19]. Yates et al. observed PSMs in two patients with morbid obesity (13%) with pT3 disease, which was slightly lower than in the non-obese group (17.5%) [20]. In our study, we did not find any significant difference in PSM or BCR. However, we found significantly higher rates of pT3a in the morbidly obese group compared to the control group. Several factors may explain this: men with obesity frequently have elevated serum levels of insulin, IGF-1, and leptin, with reduced adiponectin levels—all of which have been associated with prostate cancer in some studies [3]. Furthermore, lower serum testosterone in obese men has been linked to an increased risk of aggressive prostate cancer [34].

The current literature on the impact of morbid obesity on potency is limited, as most studies provide only minimal discussion on this outcome. Haidar et al. did not address potency in detail. Still, they observed that nerve-sparing procedures were more challenging in patients with morbid obesity, with the procedure classified as ‘very difficult’ in 30.3% of the morbidly obese group compared to 14.6% in the non-morbidly obese group [19]. Yates et al. also did not provide detailed outcomes on potency but mentioned lower erectile function rates in the obese group compared to patients without obesity [20]. Han et al. also did not delve into erectile function outcomes in detail but indicated that morbid obesity was linked to an increased risk of postoperative complications, which could potentially impact recovery and potency [18]. Although we did not observe a difference in cumulative potency rates between the two groups, we did notice that the rates of nerve sparing were lower in the patients with morbid obesity because the accumulation of periprostatic fat made it harder to visualize and access the neurovascular bundles, which are critical for erectile function preservation. It is also important to mention that in addition to the nerve-sparing degree, potency outcomes are affected by several pre-operative variables, such as age, and pre-surgery erectile function status. In our study, both groups had an element of erectile dysfunction pre-operatively with a median SHIM of 16 and 17 in patients with morbid obesity and those without obesity, respectively, which may explain the relatively low rates of erectile function recovery.

Obesity has been associated with worse continence outcomes, as shown by different studies [17,28]. Yang et al. reported that patients without obesity exhibited significantly better urinary function at both 6 and 12 months post-surgery compared to patients with obesity, suggesting faster recovery in the non-obese group [28]. Similarly, a meta-analysis by Wei et al. found that obesity increased the risk of long-term urinary incontinence, particularly after RARP, with significant differences in continence rates between obese and non-obese groups at both 12 and 24 months post-surgery [17]. Similar to potency outcomes, the literature lacks comprehensive and detailed continence outcomes in patients with morbid obesity. Yates et al. reported similar continence outcomes between patients with morbid obesity and patients without obesity six months after operation, although they did not specify the measurement methods. In our study, we found no significant difference in continence rates between patients with morbid obesity and those with normal-range BMIs, but continence recovery is usually slower due to lower compliance with postoperative physical exercises and pelvic muscle therapy. Other studies did not explicitly address continence rates [19,21].

This study has several limitations. As a retrospective analysis, it is inherently susceptible to selection bias. Although propensity score matching was used to account for confounding factors, some residual confounding may persist. Additionally, the reliance on a single institution and a single high-volume surgeon may limit the generalizability of our findings. As one of the highest-volume centers globally for robotic-assisted radical prostatectomy, our institution often serves as a referral center for complex cases that other surgeons may opt not to perform, such as those involving patients with a BMI > 40 kg/m^2^. This concentrated experience likely enhances our surgical outcomes, but it also introduces potential selection bias and reduces the applicability of our results to lower-volume centers or less experienced surgeons. Nevertheless, this study includes the largest cohort of patients with morbid obesity undergoing RARP to date, enabling a robust analysis of the impact of morbid obesity on functional and oncological outcomes while addressing an important gap in the current literature.

## 5. Conclusions

Our study provides the largest analysis to date on the impact of morbid obesity (BMI > 40 kg/m^2^) on robotic-assisted radical prostatectomy (RARP). We found that patients with morbid obesity experienced longer operative times, increased estimated blood loss, and reduced nerve-sparing rates compared to normal-weight patients. However, despite these intraoperative challenges, functional and oncological outcomes, including biochemical recurrence and continence rates, remained comparable between groups.

These findings suggest that RARP remains a safe and feasible surgical option for patients with morbid obesity when performed at a high-volume center by experienced robotic surgeons. The absence of significant differences in oncological and functional outcomes challenges previous concerns regarding surgical feasibility and cancer control in this population.

Our results align with prior studies, which have shown increased intraoperative complexity in patients with obesity but no major impact on oncological control. However, our study is unique in utilizing propensity score matching to minimize confounding, strengthening the validity of these findings.

Given the rising prevalence of morbid obesity worldwide, further multi-institutional prospective studies are needed to validate these findings and explore additional factors that may influence long-term survival and functional recovery in this high-risk population.

## Figures and Tables

**Table 1:** Comparison of preoperative patient characteristics in the study groups after 1:1 propensity score (PS) matching reporting the median value with the interquartile range (IQR) and the number of patients with the percentage; standardized mean difference evaluates the degree of covariate imbalance. PSA (Prostate Specific Antigen), BMI (Body Mass Index), SHIM (Sexual Health Inventory for Men), AUA (American Urological Association), ISUP (International Society of Urological Pathology).

Parameters	BMI (18.5–24.9 kg/m^2^) (n = 183)	BMI > 40 kg/m^2^ (n = 183)	P	Standardized Mean Difference After 1:1 PS Matching
Age	61	60	0.8	−0.001
(years)	(54–67)	(55–65)		
PSA	5.4	5.7	0.1	0.088
(ng/mL)	(4.1–8.2)	(4.5–8.9)		
Diabetes (n, %)			1	
No	119 (65)	118 (64.48)		0.011
Yes	64 (35)	65 (35.52)		−0.011
Dyslipidemia (n, %)			1	
No	89 (48.63)	90 (49.18)		−0.011
Yes	94 (51.37)	93 (50.82)		0.011
HTN (n, %)			0.8	
No	38 (20.77)	41 (22.40)		−0.04
Yes	145 (79.23)	142 (77.60)		0.04
History of prostate surgery (n, %)			1	
No	180 (98.36)	179 (97.81)		0.04
Yes	3 (1.64)	4 (2.19)		−0.04
Preoperative	17	16	1	0.001
SHIM	(6–24)	(6–23)		
Preoperative	8	8	1	0.021
AUA score	(3–15)	(3–15)		
Charlson Comorbidity Index (n, %)			1	
0	9 (4.92)	9 (4.92)		0
1–2	111 (60.66)	111 (60.66)		0
3–4	60 (32.79)	59 (32.24)		0.012
>4	3 (1.64)	4 (2.19)		−0.04
Biopsy ISUP grade (n, %)			0.7	
Group 1	63 (34.43)	51 (27.87)		0.142
Group 2	53 (28.96)	56 (30.60)		−0.036
Group 3	30 (16.39)	31 (16.94)		−0.015
Group 4	25 (13.66)	31 (16.94)		−0.091
Group 5	12 (6.56)	14 (7.65)		−0.043
Clinical T stage (n, %)			1	
T1	151 (82.51)	151 (82.51)		0
T2	31 (16.94)	31 (16.94)		0
≥T3	1 (0.55)	1 (0.55)		0
Follow-up (years)	5 (3–8)	5 (2–8)	0.9	0.084

**Table 2:** Comparison of intraoperative, functional, and pathological characteristics in the 1:1 propensity score-matched cohort, presenting median values with interquartile ranges (IQR) for continuous variables and absolute counts with percentages for categorical variables. EBL (Estimated blood loss), PSM (Positive Surgical Margins).

Parameters	BMI < 25 (n = 183)	BMI > 40 (n = 183)	*p*-Value
EBL (mL) EBL < 100mL (n, %) EBL > 100mL (n, %)	100 (50–100) 142 (77.60) 41 (22.40)	100 (100–200) 111 (60.66) 72(39.34)	0.001 0.001
Console time (minutes)	75 (75–80)	90 (75–90)	<0.001
Catheter time (days)	5 (4–6)	5 (4–6)	0.7
Hospitalization (days)	1 (1–1)	1 (1–1)	0.9
Pathological prostate weight (grams)	48 (41–61)	54 (44–64)	0.01
Postoperative ISUP grade (n, %)			0.2
Group 1	46 (25.14)	33 (18.03)	
Group 2	73 (39.89)	66 (36.07)	
Group 3	30 (16.39)	42 (22.95)	
Group 4	15 (8.20)	13 (7.10)	
Group 5	19 (10.38)	29 (15.85)	
Postoperative Complications			0.1
(Clavien–Dindo) (n, %)			
<III	43 (100)	58 (98.31)	
>3	0 (0)	1 (1.69)	
Lymph Node Dissection (n, %)			0.44
No	62 (33.88)	70 (38.25)	
Yes	121 (66.10)	113 (61.75)	
Pathological T stage (n, %)			0.11
pT2	117 (63.93)	101 (55.19)	
≥pT3	66 (36.07)	82 (44.81)	
pT3a	37 (20.22)	63 (34.43)	0.015
Lymphocele (n, %)			0.021
No	172 (93.99)	158 (86.34)	
Yes	11 (6.01)	25 (12.66)	
Nerve-sparing degree (n, %)			<0.001
None	9 (4.92)	9 (4.92)	
Partial	103 (56.28)	143 (78.14)	
Full	71 (38.80)	31 (16.94)	
PSM (n, %)			0.3
No	149 (81.42)	139 (75.96)	
Yes	34 (18.58)	44 (24.04)	

**Table 3:** Cumulative 12-month and 24-month rates and estimated hazard ratios (with 95% confidence intervals) for continence recovery, potency recovery, and biochemical recurrence (BCR).

Parameters	BMI		*p*
	BMI < 25	BMI > 40	
Potency			
12 mo. cumulative rate (%)	35.5 (29.0–43.0)	36.0 (29.6–43.5)	0.9
24 mo. cumulative rate (%)	43.4 (36.5–51.0)	43.7 (36.9–51.3)	
HR (95% CI)	reference	0.97 (0.72–1.32)	
Continence			
12 mo. cumulative rate (%)	84.0 (78.5–89.0)	80.3 (74.2–85.7)	0.9
24 mo. cumulative rate (%)	86.0 (80.5–90.6)	89.1 (84.0–93.1)	
HR (95% CI)	Reference	1 (0.81–1.26)	
BCR			
12 mo. cumulative rate (%)	3 (1.1–6.4)	3.8 (1.8–7.8)	0.2
24 mo. cumulative rate (%)	5 (2.7–9.6)	6.7 (3.8–11.4)	
HR (95% CI)	Reference	1.52 (0.79–2.9)	

## Data Availability

The authors confirm that the data supporting the findings of this study are available within the article. Raw data that support the findings are available from the corresponding author (AJ) upon reasonable request.

## Supplementary Materials

The following supporting information can be downloaded at: https://www.mdpi.com/article/10.3390/cancers17040709/s1, Figure S1: Flow Diagram for inclusion and exclusion criteria.
