# Supplementary figures and images for: Impact of Class III Obesity (Morbid Obesity) on the Perioperative, Functional, and Oncological Outcomes of Robotic-Assisted Radical Prostatectomy
Source: Cancers (Basel). 2025 Feb 19;17(4):709. doi: 10.3390/cancers17040709 (PMC11853698; doi:10.3390/cancers17040709)

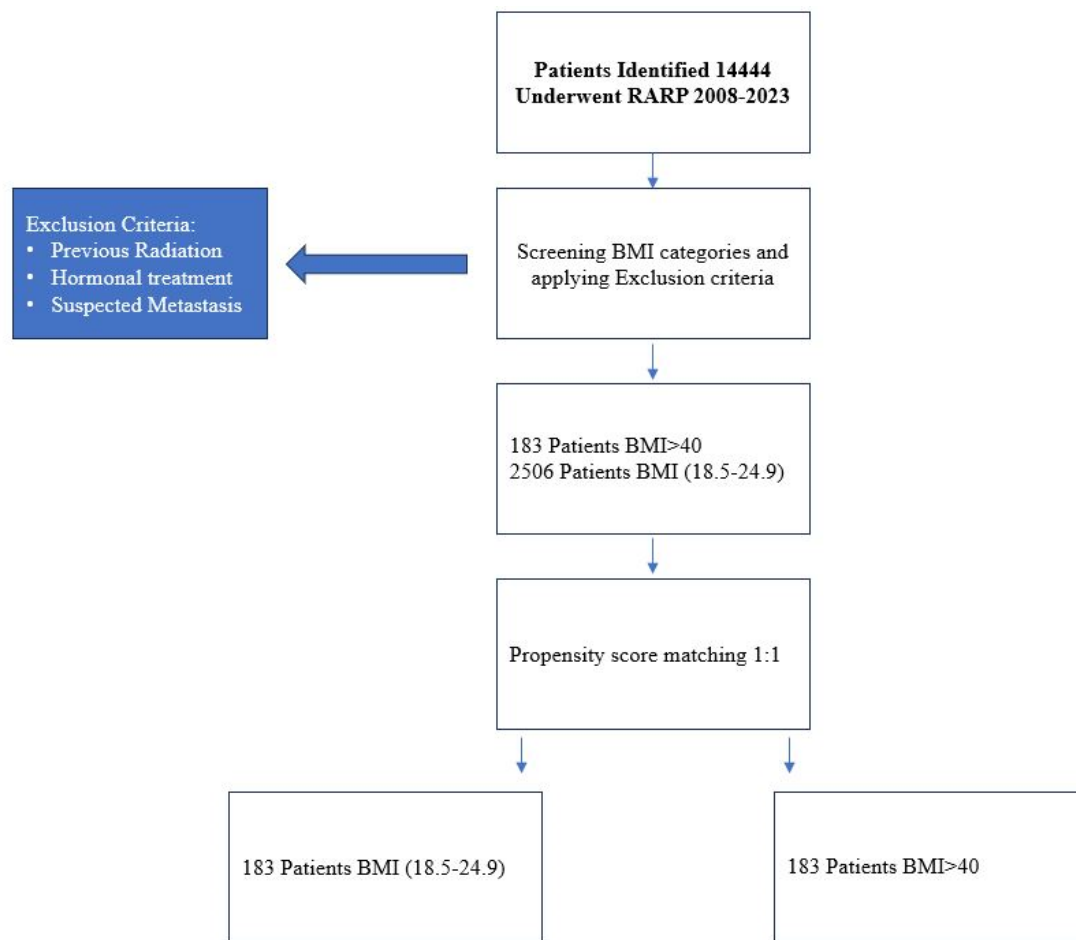

Figure S1: Flow Diagram for inclusion and exclusion criteria

Supplement: Supplementary file 1 [file cancers-17-00709-s001.zip › cancers-3454060-supplementary.pdf]
